# Low-Cycle Fatigue Life Prediction of Pure Copper Based on Slip Deformation Inhomogeneity Analysis

**DOI:** 10.3390/ma19194220

**Published:** 2026-10-04

**Authors:** Lili Jin, Shenghuan Qin, Hai Wu, Keshi Zhang

**Affiliations:** 1 School of Municipal Construction and Transportation, Guangxi Polytechnic of Construction, Nanning 530007, China; 2 School of Management Science and Engineering, Guangxi University of Finance and Economics, Nanning 530003, China; 3 Key Lab of Disaster Prevention and Structural Safety, College of Civil Engineering and Architecture, Guangxi University, Nanning 530004, China

**Keywords:** low-cycle fatigue, life prediction, T2 pure copper, polycrystal, deformation inhomogeneity, crystal plasticity

## Abstract

Tension-compression strain fatigue tests and observations on the mesoscopic evolution of deformation inhomogeneity were conducted on T2 pure copper. By combining the representative volume element (RVE) with the crystal plasticity finite element method (CPFEM), the entire processes of grain-level inhomogeneous deformation of the specimens under fatigue cycles at seven strain amplitudes were simulated. On this basis, the parameters characterizing the deformation inhomogeneity and their correlation with material fatigue were investigated. Three sets of fatigue indicator parameters (FIPs) based on slip deformation inhomogeneity were proposed: (1) the mean value $\bar{\Gamma }$ and standard deviation $\hat{\Gamma }$ of the sum of the absolute values of the resolved shear strains of the slip systems at the tensile peak point; (2) the standard deviation $\hat{\omega }$ of the sum of the products of the resolved shear strains and resolved shear stresses of the slip systems; (3) the mean value $\bar{\Xi }$ and standard deviation $\hat{\Xi }$ of the product of the sum of the absolute values of the resolved shear strains and the sum of the absolute values of the resolved shear stresses of the slip systems. These three sets of FIPs can be utilized for fatigue life prediction in material testing. Error evaluation confirms that the predicted fatigue lives obtained using this method all fall within a factor of 1.5 of the experimental values.

## 1. Introduction

Fatigue failure is a prevalent form of material and structural degradation in engineering. The vast majority of mechanical components and engineering structures are subjected to cyclic loading. Statistics indicate that over 80% of component damage and structural failures are fatigue-related. Under cyclic loading, some components with intact appearances and no obvious plastic deformation may experience sudden fatigue fracture, leading to catastrophic consequences. Therefore, it is highly necessary to conduct fatigue tests on engineering materials and explore methods for predicting fatigue life [1,2,3].

Currently, the most widely used methods for evaluating fatigue properties of materials are empirical statistical methods based on extensive experimental data. These include the Basquin equation, which describes the relationship between stress amplitude and fatigue life [4], as well as the Manson-Coffin equation, which defines the relationship between plastic strain amplitude and fatigue life [5,6], along with its modified forms [7,8]. To date, these experiment-based life prediction methods are still widely used. Researchers have extended them to predict the fatigue life of various engineering components under diverse operating conditions, continuously improving them to enhance prediction accuracy and efficiency [9,10,11]. These improvements encompass considerations of energy dissipation and the influence of material microstructures. Since the fatigue process is inherently accompanied by energy accumulation and dissipation, many scholars have adopted energy or its related quantities as criteria for life prediction. Macroscopic energy analysis achieves fatigue life prediction by calculating energy from hysteresis loops or applying the principle of entropy increase [12,13,14,15,16,17]. Such methods have also been applied to evaluate the fatigue life of various composite materials [18,19,20] and the creep-fatigue life [21,22,23]. Although these phenomenological models are convenient for engineering applications, they lack a physical description of the internal microscopic deformation mechanisms of materials, and thus cannot reveal the physical essence of fatigue damage. Due to the absence of a universally applicable physical foundation, whenever the material’s microstructure (e.g., grain size, texture, impurity phases) or loading conditions change, extensive re-testing is required to recalibrate the empirical parameters.

With the deepening understanding of fatigue mechanisms, the academic community has recognized that the fatigue failure of metallic materials is closely related to the accumulation of local damage induced by dislocation slip. This damage exhibits strong inhomogeneity at the grain scale and typically initiates at slip bands or grain boundaries of grains with specific orientations [24,25]. To reveal this process from the perspective of physical mechanisms, the crystal plasticity finite element method (CPFEM) based on mesoscopic mechanical analysis has been widely applied [26,27]. Compared to macroscopic phenomenological models, the crystal plasticity approach can directly simulate complex crystal slip within materials, taking into account anisotropy at small scales, texture evolution, and inter-granular interactions [28,29,30]. By constructing a representative volume element (RVE), this method can “see” the microscopically inhomogeneous stress and strain fields under macroscopically uniform stress, thereby capturing the driving force for crack initiation at the mesoscopic scale [31]. Manonukul et al. utilized RVEs combined with crystal plasticity analysis to demonstrate that the low-cycle fatigue life of metallic materials can be predicted, and that the critical accumulated slip can serve as a criterion for crack initiation [32]. Sweeney et al. conducted a crystal plasticity analysis on fatigue crack initiation in metallic notched specimens under low-cycle loading, indicating that the crack initiation sites are correlated with the equivalent plastic strain and accumulated plastic slip [33]. Castiglioni et al. [34] pointed out that under cyclic loading, the failure of steel components is closely related to cumulative damage models, laying a foundation for understanding the physical mechanisms of fatigue crack initiation. To quantify this damage at the grain scale, researchers have introduced fatigue indicator parameters (FIPs). Li et al. [35] demonstrated in their study on AA7075-T6 aluminum alloy that calculating FIPs via CPFEM combined with extreme value statistics theory can effectively capture the scatter in fatigue life from high-cycle to ultra-high-cycle regimes. To overcome the computational cost caused by microstructural randomness, Gu et al. [36] further proposed a statistical framework based on extreme value theory, enabling the reliable prediction of maximum FIPs within bulk materials by simulating a limited number of statistical volume elements (SVEs). However, the distribution of FIPs and crack initiation sites is not solely determined by statistical laws but is also heavily constrained by local microstructural features. In their simulations of Al6082-T6, Hallberg et al. [37] found that microstructural features such as twin boundaries act as significant obstacles to plastic slip, and the resulting local damage may even exceed the effect of surface roughness. This indicates that predicting crack initiation requires a comprehensive consideration of both statistical extremes and microstructural heterogeneity.

Zhang et al. combined crystal plasticity constitutive models with RVEs to investigate the evolution of subsequent yield surfaces in metallic materials, confirming that plastic deformation under cyclic loading significantly influences the directional evolution of yield surfaces [38,39]. Building upon this foundation, simulations of the mechanical behavior of materials during fatigue cycling revealed that the fatigue life of material specimens can be predicted using the critical values of parameters characterizing mesoscopic deformation inhomogeneity. This approach is applicable to symmetric cyclic tension-compression [40,41,42] and torsion tests [43], as well as to asymmetric axial cyclic tests [44]. However, there is currently no consensus on the superiority of the proposed FIPs, as they have only been validated under a limited range of testing conditions. Furthermore, detailed investigations into the cumulative effects of plastic slip, inhomogeneous evolution characteristics, and their combined influence with stress states during the fatigue process of polycrystalline metallic materials remain insufficient.

To further investigate these issues, this paper examines FIPs and fatigue life prediction methods based on the statistics of grain-scale inhomogeneous deformation. Section 2 introduces the strain fatigue tests on pure copper and the observations of grain-scale inhomogeneous slip deformation. Section 3 presents the crystal plasticity constitutive model, the RVE, and the calibration of model parameters adopted in this study. Section 4 discusses the FIPs derived from the statistical analysis of inhomogeneous slip deformation, applies them to predict the fatigue life of pure copper, and performs an error analysis. Section 5 summarizes the main conclusions of this paper. Finally, Section 6 outlines the limitations of this study and provides perspectives for future research. 

## 2. Experimental Investigation of Pure Copper

### 2.1. Material Properties of Pure Copper

**Figure 1:**
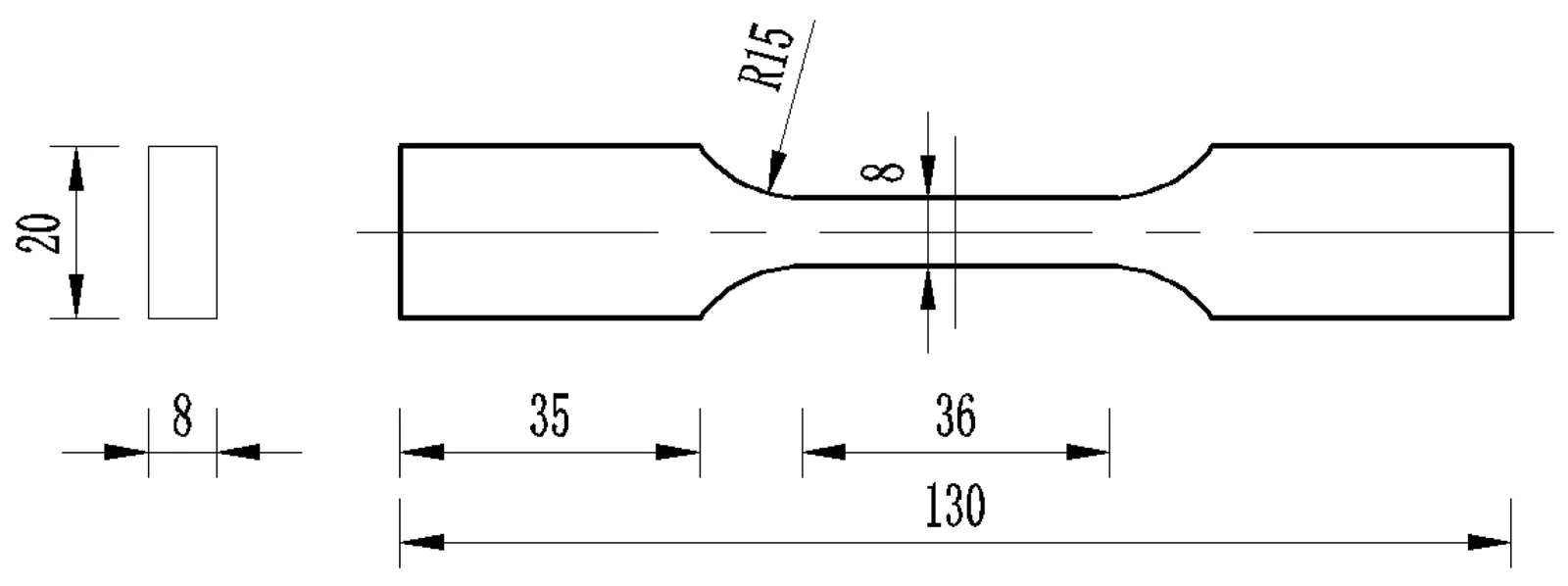
Geometric dimensions of the pure copper specimen [46].

**Figure 2:**
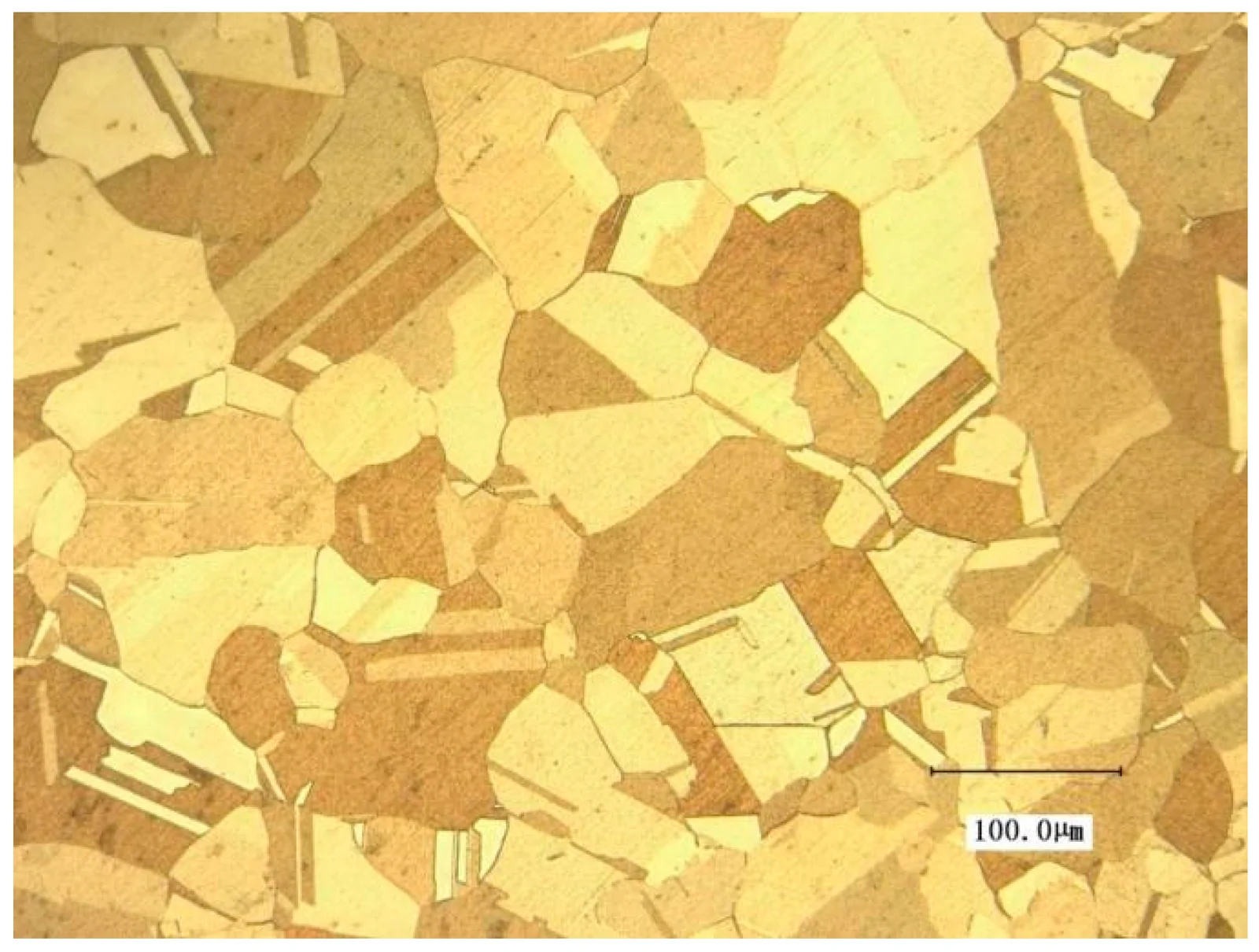
Metallographic microstructure of T2 pure copper.

**Figure 3:**
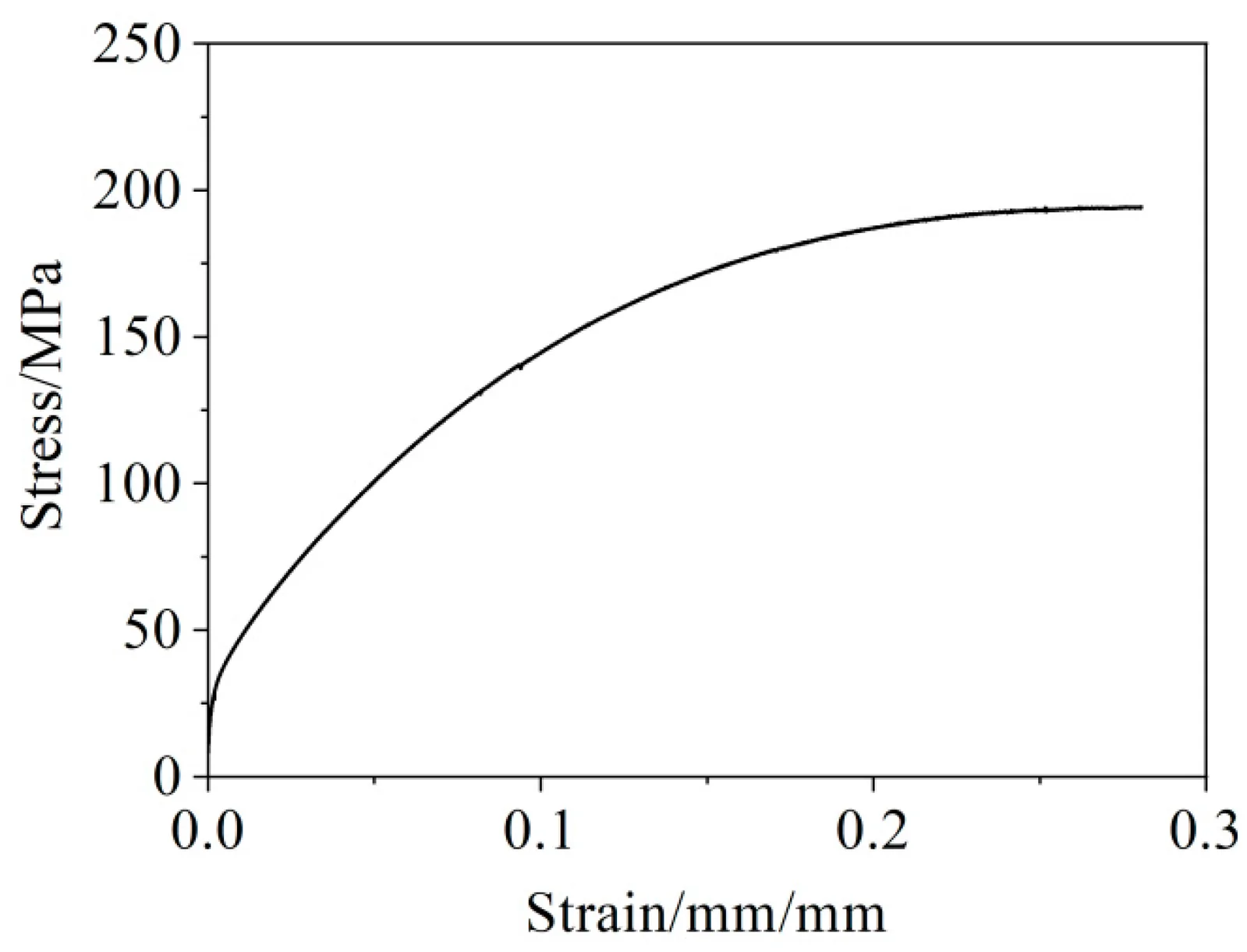
Stress–strain curve of pure copper under uniaxial tension.

The test material used in this study was industrial pure copper T2, supplied as a bar with a diameter of 26 mm. The average grain size was approximately 55 μm, and the material density was 8.96 g/cm^3^. The chemical composition is presented in Table 1, and the specific dimensions of the specimens are illustrated in Figure 1. The metallographic microstructure of the material was observed through grinding, polishing, and chemical etching, as shown in Figure 2. Uniaxial tensile tests were conducted to evaluate the fundamental mechanical properties of the material, yielding the uniaxial stress–strain curve of the pure copper. The basic mechanical properties are summarized in Figure 3 and Table 2, respectively.

### 2.2. Strain Fatigue Tests of Pure Copper

**Figure 4:**
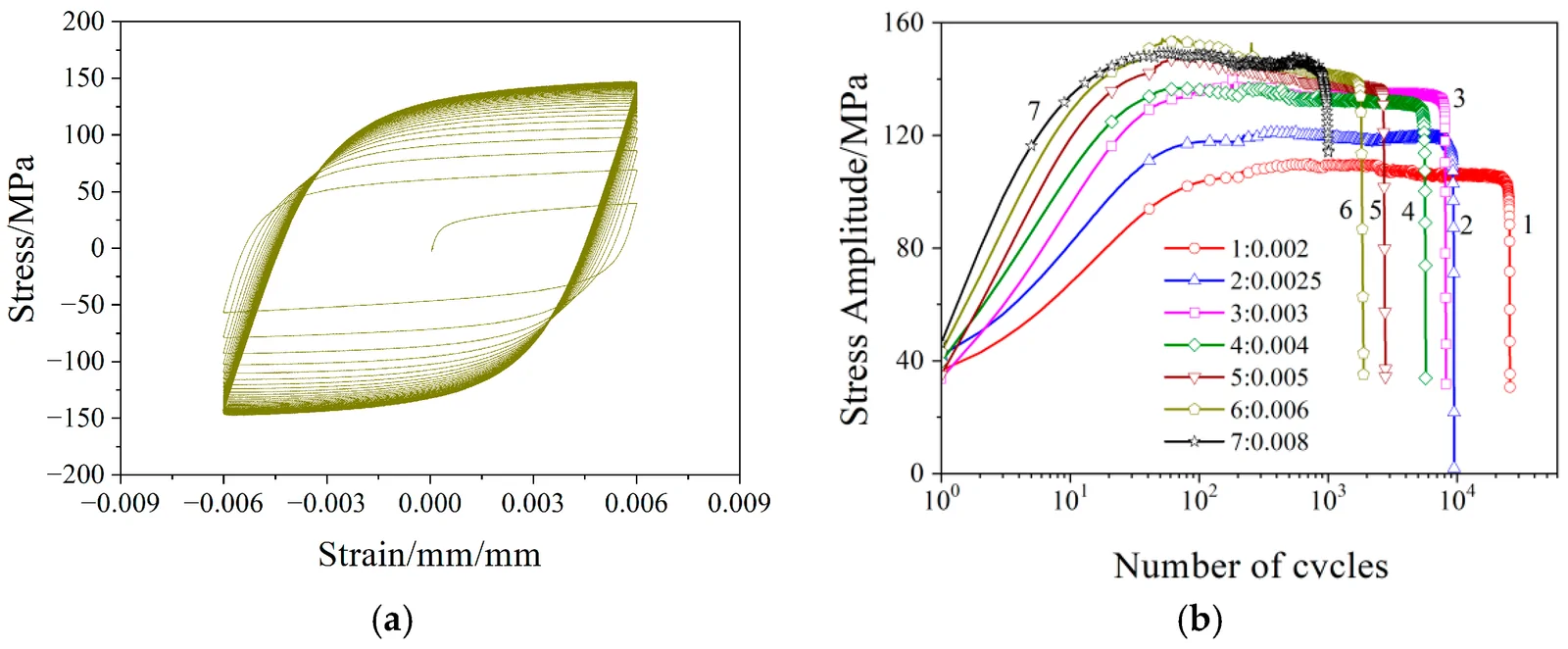
Cyclic hardening phenomenon of pure copper. (**a**) Stress–strain curves before stabilization at a strain amplitude of 0.006; (**b**) Variation in peak stress with the number of cycles for pure copper.

**Figure 5:**
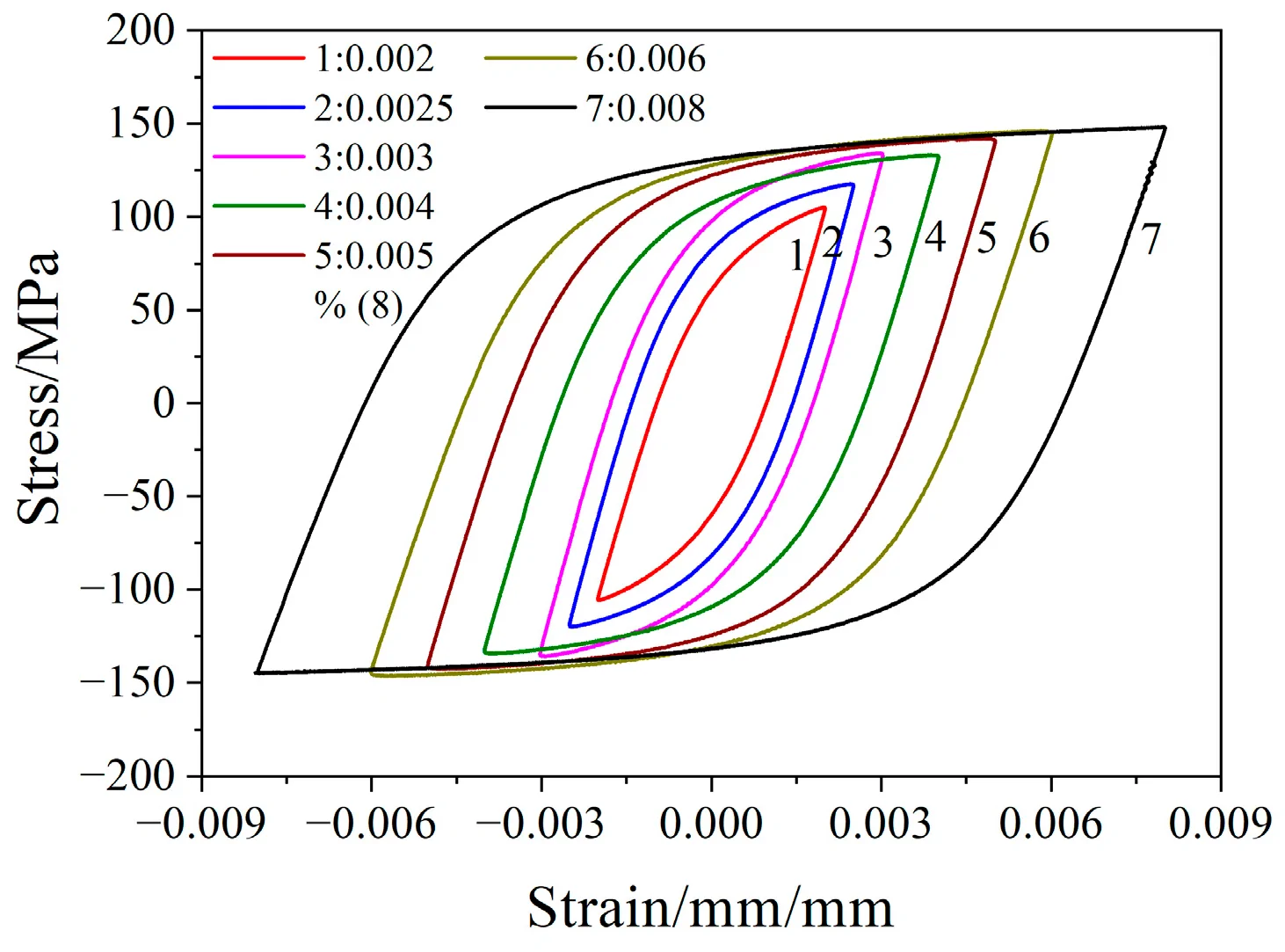
Stable hysteresis loops of T2 pure copper under different strain amplitudes.

Both the mechanical property tests and fatigue tests of pure copper were conducted in the Key Laboratory of the Ministry of Education at Guangxi University. The testing equipment used was an MTS 809.25 electro-hydraulic servo tension-torsion testing machine (MTS Systems Corporation, Eden Prairie, MN, USA). The fatigue tests were performed at room temperature under strain control with a strain ratio of *R_ε_* = −1. The controlled strain amplitudes *E_a_* were set to 0.002, 0.0025, 0.003, 0.004, 0.005, 0.006, and 0.008. A triangular waveform was applied at a frequency of 0.5 Hz. The fatigue life was defined as the number of cycles to failure, i.e., the cycle at which a macroscopic crack appeared on the specimen surface. At least two specimens were tested for each strain amplitude. The test results were considered valid if the difference between the measured fatigue lives was within a factor of 2 [47]. The material exhibited significant cyclic hardening during the initial loading stage (within the first 100 to 200 cycles), followed by a stable cyclic state. Stress–strain curves before stabilization at a strain amplitude of 0.006 is shown as Figure 4a. The variation in the peak stress in the hysteresis loops with the number of cycles is shown in Figure 4b. After several dozen cycles, both the hysteresis loops and the peak stresses stabilized with respect to the cycle number. Therefore, the stable hysteresis loops were extracted at the 200th, 200th, 80th, 45th, 45th, 80th, and 80th cycles for the respective strain amplitudes (from low to high), as plotted in Figure 5. These stable hysteresis curves were subsequently used to calibrate the parameters of the crystal plasticity constitutive model. The low-cycle fatigue life data obtained from the tests are summarized in Table 3.

**Figure 6:**
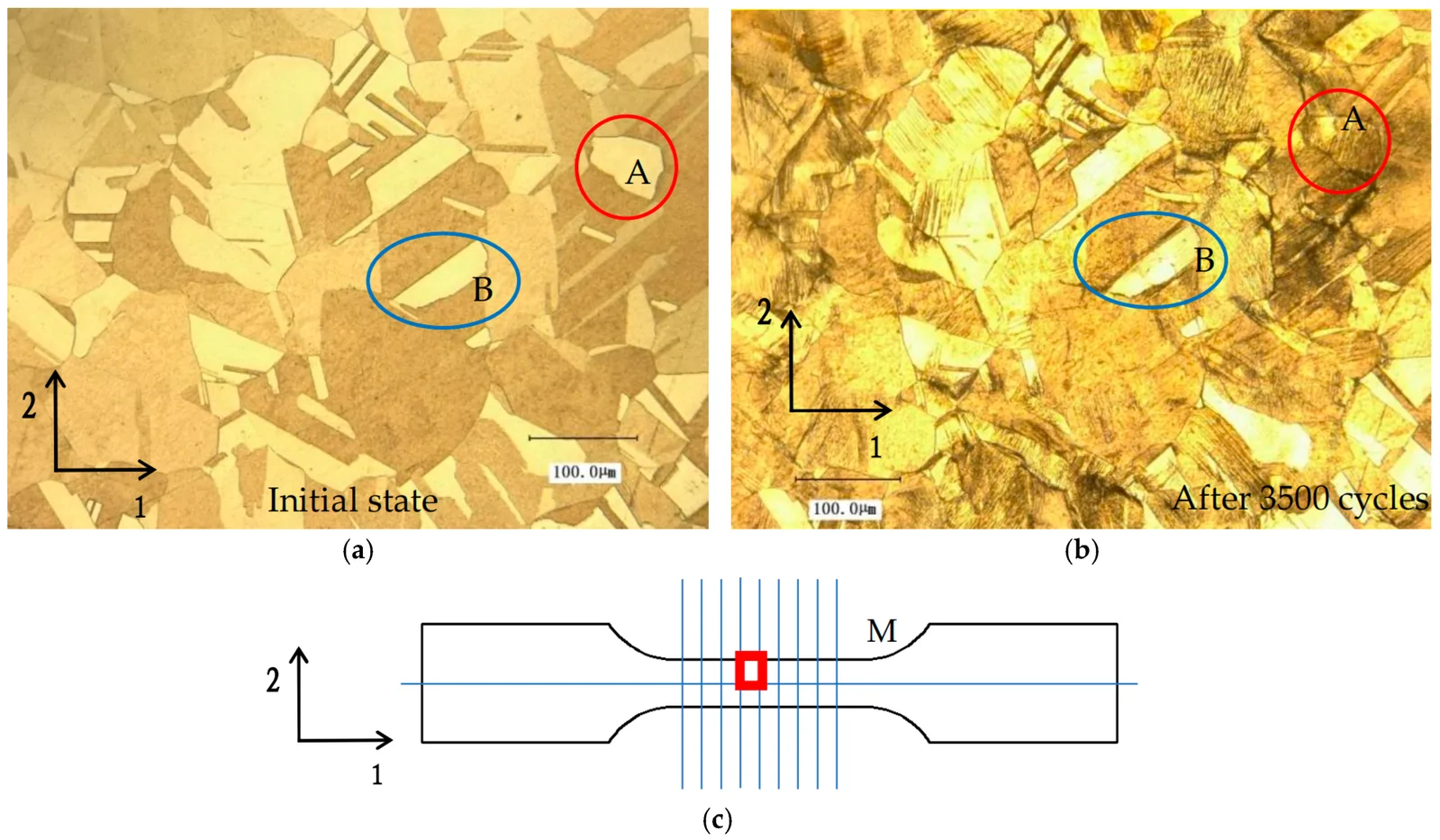
Surface morphology at zone M of the specimen under a strain amplitude of 0.003: (**a**) Surface morphology of the specimen in the observed region at the initial state, before loading; (**b**) Surface morphology of the specimen in the observed region after 3500 cycles (The loading direction is along the positive direction of Axis 1); (**c**) Location of the observed region on the specimen (zone M).

For the fatigue test with a strain amplitude of 0.003, metallographic observations were conducted on the surface of the gauge section (Zone M) of the specimen to investigate the deformation differences among individual grains under macroscopically uniform deformation. Metallographic images obtained at the initial state and after 3500 cycles are presented in Figure 6. It can be observed that after 3500 cycles, the distribution of slip bands within the material becomes highly inhomogeneous. As shown in Figure 6b, significant differences in both the density and orientation of slip deformation can be seen among different grains. For instance, slip bands are densely distributed in Grain A, whereas they are not obvious in Grain B. These metallographic observations indicate that the deformation of polycrystalline metallic materials under cyclic loading is highly inhomogeneous at the grain scale. This inhomogeneity is evident not only across the entire observed area but also at the grain scale or even smaller scales. This implies that such grain-level deformation inhomogeneity requires statistical analysis for a more in-depth investigation. However, limited by experimental observation conditions, this deformation inhomogeneity is only reflected in surface observations and can serve merely as a qualitative reference. To obtain a more comprehensive deformation morphology of the material at the grain scale and subsequently conduct quantitative analysis, numerical simulation methods are essential.

## 3. Crystal Plasticity Constitutive Model and Representative Volume Element (RVE)

The mesoscopic inhomogeneous deformation of polycrystalline pure copper observed during strain fatigue in the preceding section is difficult to quantify statistically through experiments alone. However, numerical simulation offers a viable approach to achieve this goal. At the grain scale, the local deformation states of the material vary significantly depending on the lattice orientation. Crystal plasticity analysis can be employed to track the grain-scale deformation evolution of the material under cyclic loading. For pure copper, the grains possess a face-centered cubic (FCC) lattice structure, and their size, shape, and initial orientations are governed by the microscopic random processes during material manufacturing. At the grain scale, both the elastic and plastic behaviors of the material (which is primarily dominated by slip mechanisms) are anisotropic.

### 3.1. Crystal Plasticity Constitutive Model

According to continuum mechanics, the relationship between the Eulerian velocity gradient tensor **L**, the deformation rate tensor **D**, and the spin tensor **W** for a material point within a grain is given by:

(1) $$\begin{array}{l} D=D^{*}+D^{P}=\mathrm{sym}\left(L^{*}\right)+\mathrm{sym}\left(L^{P}\right),\\ W=W^{*}+W^{p}=\mathrm{asym}\left(L^{*}\right)+\mathrm{asym}\left(L^{P}\right) \end{array}$$

where D^*^ and **D***^P^* are the elastic and plastic deformation rate tensors, respectively, and W^*^ and **W***^p^* are the elastic and plastic spin tensors, respectively.

In the case of small elastic deformations, the rate constitutive relation can be expressed as:

(2) $${\dot{\sigma }}^{J}=\overset{\langle 4\rangle }{C}:D^{*}=\overset{\langle 4\rangle }{C}:\left(D-D^{P}\right)$$

In the formula, $\overset{<4>}{C}$ is the fourth-order elastic constitutive tensor of the crystal in the global coordinate system. The global coordinate system is fixed during the calculation process, while the crystal axis coordinate system for the crystal in each grain is calculated according to its orientation and rotation history; ${\dot{\sigma }}^{J}$ is the Jaumann stress rate, which excludes the effect of rigid-body rotation during the deformation process, and

(3) $${\dot{\sigma }}^{J}=\dot{\sigma }-W\cdot \sigma +\sigma \cdot W$$

where **σ** and $\dot{\sigma }$ are the Cauchy stress tensor and its rate, respectively. Over the time interval *t* to *t*+Δ*t*, integrating the deformation rate **D** yields the logarithmic strain increment Δ**ε**. Using Equations (1) and (2), the incremental accumulation of **σ** can be performed as follows:

σt+Δt−σt+Δtt=ΔσJ

(4) or σt+Δt=σt+Δtt+C<4>:Δε−Δεp

In the formula, Δ**ε***^p^* is the logarithmic plastic strain increment, and σt+Δtt is *^t^***σ** in the configuration at time *t*+Δ*t*.

For plastic deformation under the slip mechanism, the relationship between the resolved shear stress and the resolved shear strain rate of the *α*-th slip system can be described by introducing a back stress term. This is based on the evolution law of slip resolved shear strain for single crystals proposed by Hutchinson [48], drawing on the Chaboche model [41,42,43] to account for the Bauschinger effect.

(5) $${\dot{\gamma }}^{\left(\alpha \right)}={\dot{\gamma }}_{0}\mathrm{sgn}\left(\tau^{\left(\alpha \right)}-x^{\left(\alpha \right)}\right){\left|\frac{\tau^{\left(\alpha \right)}-x^{\left(\alpha \right)}}{g^{\left(\alpha \right)}}\right|}^{k}$$

where *τ*^(*α*)^ and *x*^(*α*)^ are the resolved shear stress and the corresponding back stress of the *α*-th slip system of the single crystal, respectively; *g*^(*α*)^ is a scalar function describing the elastic range of the shear stress for the *α*-th slip system; ${\dot{\gamma }}^{\left(\alpha \right)}$ is the reference shear strain rate, which is a material constant to be determined; *k* is a material constant reflecting the strain rate sensitivity of the material. The evolution of the back stress *x*^(*α*)^ is described by the following equation [49]:

(6) $${\dot{x}}^{\left(\alpha \right)}=a{\dot{\gamma }}^{\left(\alpha \right)}-c\left[1-e_{1}\left(1-\exp\left(-e_{2}\gamma \right)\right)\right]x^{\left(\alpha \right)}\left|{\dot{\gamma }}^{\left(\alpha \right)}\right|-px^{\left(\alpha \right)}$$

In the equation, *a* is a material constant describing the linear hardening of slip systems, *c* and *p* are material constants reflecting the nonlinear kinematic hardening characteristics, and *e*_1_ and *e*_2_ are material constants reflecting the cyclic hardening (softening) characteristics. The introduction of back stress enables the crystal plasticity constitutive model to describe the hardening (softening) behavior of the material during cyclic loading.

The evolution of the scalar function *g*^(*α*)^ is calculated using the formula suggested by Pan and Rice [50]:

(7) $${\dot{g}}^{\left(\alpha \right)}\left(\gamma \right)=\sum_{\beta =1}^{n}h_{\alpha \beta }\left(\gamma \right)\left|{\dot{\gamma }}^{\left(\beta \right)}\right|,\gamma =\int \sum_{\beta =1}^{n}\left|d\gamma^{\left(\beta \right)}\right|$$

where *h_αβ_* is the slip hardening modulus, and *n* denotes the total number of slip systems. Hutchinson [51] suggested that this modulus be calculated according to the following formula:

*h_αβ_*(*γ*) = *h*(*γ*)[*q* + (1 − *q*)*δ_αβ_*] (8)

where *q* is a constant. Chang and Asaro [52] suggested calculating it using the following formula:

(9) $$h\left(\gamma \right)=h_{0}{\mathrm{sech}}^{2}\left(\frac{h_{0}\gamma }{\tau_{s}-\tau_{0}}\right)$$

In the equation, *h*_0_ is the initial hardening rate, and *τ*_0_ and *τ_s_* are the critical resolved shear stresses at the initial stage and after saturation, respectively, which are all considered as material constants. All parameters of the above model have definite physical meanings with respect to the stress–strain hysteresis loops, and can be determined through measured curves combined with iterative numerical simulations.

The initial unit normal vector of the slip plane of the *α*-th slip system and the initial unit direction vector of the *α*-th slip system are denoted as **n**^(*α*)^ and **m**^(*α*)^, respectively. Following the treatment approach of previous scholars [53,54], the Schmid tensor reflecting the relationship between the slip system shear strain and shear stress and the strain and stress in the Cartesian global coordinate system is:

(10) $$P^{\left(\alpha \right)*}=\frac{1}{2}\left(m^{\left(\alpha \right)*}n^{\left(\alpha \right)*}+n^{\left(\alpha \right)*}m^{\left(\alpha \right)*}\right),\;m^{\left(\alpha \right)*}=F^{*}\cdot m^{\left(\alpha \right)},\;n^{\left(\alpha \right)*}=n^{\left(\alpha \right)}\cdot F^{*-1}$$

where F^*^ is the elastic part of the deformation gradient tensor. The incremental logarithmic strain tensor in Equation (3) can be calculated as follows [55]:

(11) $$\Delta \varepsilon^{p}=\sum_{\alpha =1}^{n}P^{\left(\alpha \right)*}\Delta \gamma^{\left(\alpha \right)}$$

where Δ*γ*^(*α*)^ is obtained by integrating ${\dot{\gamma }}^{\left(\alpha \right)}$. Furthermore, according to the Schmid law, we have:

*τ*^(*α*)^ = **P**^(*α*)∗^:**σ** (12)

The calculation process and algorithm of the above model are detailed in references [40,41,56,57], and can be implemented through the user material subroutine UMAT of the ABAQUS version 6.5 (Abaqus, Inc., Providence, RI, USA).

### 3.2. RVE for Simulating the Mesoscopic Structure of Polycrystalline Materials

**Figure 7:**
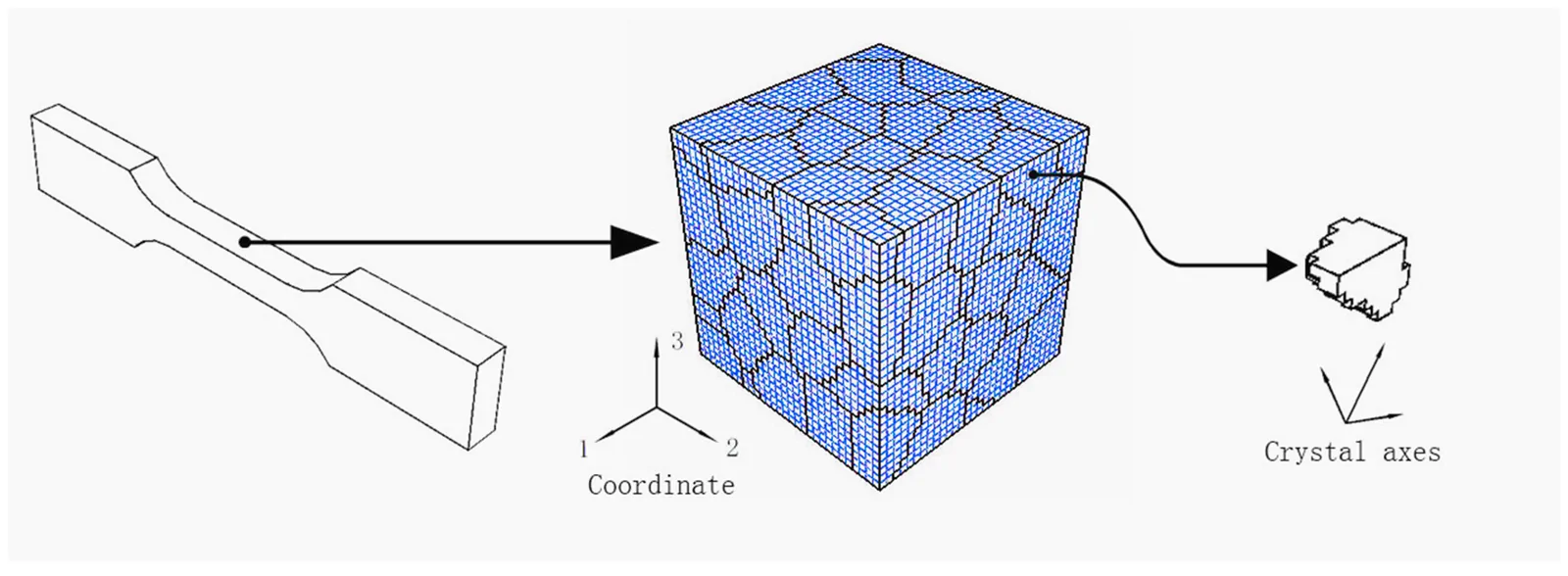
Schematic diagram of RVE. The RVE represents the material point within the gauge section of the specimen. It consists of 100 grains and 27,000 elements.

During the loading process, the macroscopic deformation within the gauge length of the specimen can be assumed to be uniform. However, due to the anisotropy at the grain scale in polycrystalline pure copper, the mesoscopic structure is highly heterogeneous, resulting in non-uniform material deformation at the grain level. To simulate the polycrystalline structure of pure copper, an RVE constructed using a Voronoi polycrystalline aggregate (as shown in Figure 7) is employed as the material model. This model takes the form of a cube with a unit edge length (without considering grain size effects) and consists of 100 randomly generated single-crystal grains (When the RVE contains 100 grains, it can be considered that the RVE size has basically achieved convergence from the perspective of describing the macroscopic mechanical behavior of the material [41]). The crystallographic orientation of each grain (direction cosines of the crystal axes) is generated using random numbers [58,59]. The grains in the model exhibit direction-dependent mechanical properties, which are described by the crystal viscoplasticity model introduced previously. Furthermore, the model accounts for the Bauschinger effect, enabling the simulation of anisotropic constitutive behavior of grains under cyclic loading. The RVE is discretized into 27,000 eight-node hexahedral elements, comprising a total of 29,791 nodes.

The mesostructure of the Representative Volume Element (RVE) was idealized using an ideal grain boundary assumption, excluding explicit metallurgical defects. This study focuses on quantifying the relationship between the FIPs and crystal plastic slip-driven deformation heterogeneity. While geometric grain boundaries were not explicitly modeled, the orientation gradients between adjacent grains generate local strain gradients and stress concentrations that capture the grain boundary constraints on slip transfer. Thus, the accumulated plastic slip-based FIP effectively identifies deformation heterogeneity for fatigue life prediction. Additionally, as the critical FIP value was calibrated against experimental fatigue life, the statistical effects of metallurgical defects were inherently absorbed into this parameter, ensuring the predictions reflect macroscopic fatigue behavior. This approach provides a reasonable trade-off, avoiding the high computational cost of explicit defect modeling while validating the FIP’s physical relevance to fatigue damage.

Consistent with the state of the specimen’s gauge section during actual strain-controlled symmetric tension-compression cyclic tests, the RVE maintains a uniform macroscopic deformation. The surface boundary conditions are defined as follows: an axial displacement in the 1-direction is applied to the positive surface normal to the 1-axis; the normal displacements are set to zero on the negative surfaces normal to the 1-, 2-, and 3-axes, respectively; on the positive surfaces normal to the 2- and 3-axes, the nodal normal displacements are constrained to be equal, respectively, while the macroscopic normal and shear stresses are set to zero. Under this loading scheme, the six faces of the RVE remain planar throughout the deformation process (indicating macroscopic uniform deformation). Meanwhile, the RVE maintains a macroscopic uniaxial stress state and satisfies the periodicity assumption. In the current calculations, the effects of metallurgical defects and grain boundaries are not considered.

### 3.3. Calibration and Validation of Crystal Plasticity Model Parameters for T2 Pure Copper

The parameters of the crystal plasticity model have a clear correspondence with the characteristics of the stress–strain hysteresis loops. Following the method proposed in Ref. [40], these parameters were calibrated based on the stable hysteresis loops obtained from the experiments. Table 4 presents the calibration results of the crystal elastic-plastic model parameters for the T2 pure copper test material.

**Figure 8:**
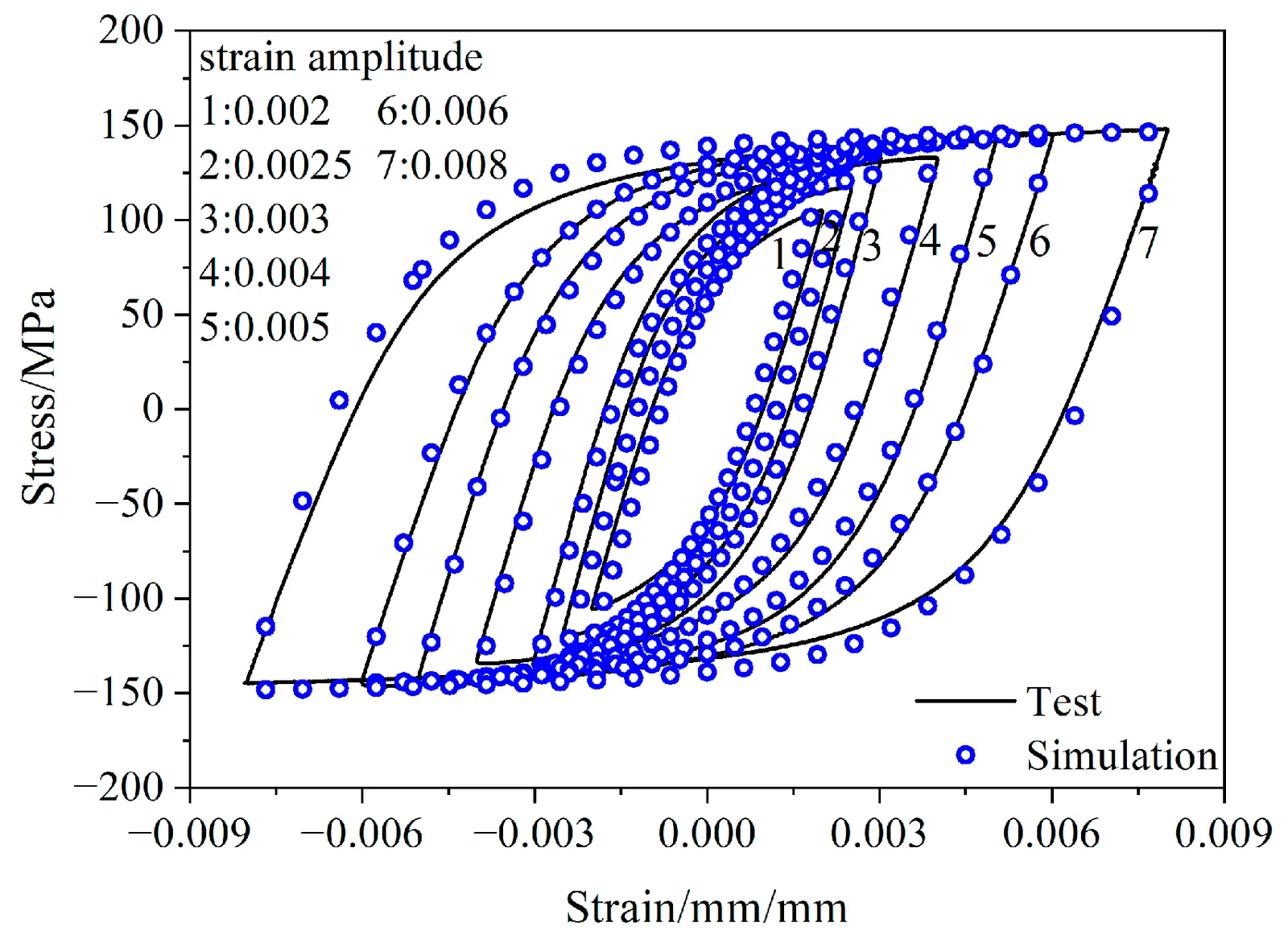
Comparison of the stable hysteresis loops of pure copper between numerical simulations and experimental measurements under different strain amplitudes.

The rationality and effectiveness of the proposed model and its parameters were verified by comparing the numerical simulations with the experimental results. Figure 8 illustrates the fitting between the calculated hysteresis loops (obtained using the RVE model in Figure 7 combined with the crystal plasticity constitutive model) and the experimentally measured hysteresis loops under strain amplitudes of 0.002, 0.0025, 0.003, 0.004, 0.005, 0.006, and 0.008. In recent years, the use of CPFEM simulations validation through fitting stress–strain curves has also been widely accepted; therefore, the excellent agreement between the calculated and experimental results confirms that the proposed model and the calibrated parameters are both reasonable and effective [30,59].

Since pure copper exhibits approximate Masing material behavior, the stable hysteresis loops at different strain amplitudes approximately follow the same evolution law. Although, in theory, parameters calibrated from a single strain amplitude could be used to predict responses at other amplitudes, in practice, we incorporated experimental data from all seven strain amplitudes during parameter calibration. This approach was adopted to eliminate potential accidental errors associated with a single data point and to enable the crystal plasticity constitutive model to provide a relatively accurate and robust description of the material’s hysteresis behavior across all seven investigated strain amplitudes. The aim of this strategy is to obtain a set of “globally optimal parameters” that represent the intrinsic material properties over the entire strain range, rather than performing a local fit tailored to a specific strain amplitude.

## 4. Fatigue Life Prediction of Pure Copper Based on Statistical Analysis of Inhomogeneous Slip Deformation

### 4.1. Fatigue Indicator Parameters (FIPs) Based on Inhomogeneous Slip Deformation

The aforementioned RVE model can be used to describe or reflect the macroscopic mechanical behavior of polycrystalline metals, as well as the heterogeneous evolution of mesoscopic deformation and stress. The macroscopic mechanical behavior is characterized by the statistical mean of the deformation and stress within the RVE, while the heterogeneous variations in mesoscopic deformation and stress are reflected by the corresponding statistical analysis parameters (i.e., mean values or standard deviations). Studies in the literature [40,41,42,43,44] have demonstrated that these parameters increase with cycling, reflecting the microstructural evolution induced by plastic deformation. Consequently, they are considered to be correlated with the fatigue damage of polycrystalline metals. The microstructural changes in materials caused by plastic deformation can be considered to be associated with the fatigue damage of polycrystalline metallic materials; if their critical values are used to predict the fatigue life of the material, the prediction errors are all within a reasonable range. Although the above-mentioned literature and early studies have recognized that slip accumulation or its related quantities can serve as FIPs, the influence of stress state on fatigue has not been considered, and the parameters γ^(α)^ have not been directly incorporated into the statistical quantities. The evolution processes of γ^(α)^ are directly driven by the cyclically varying resolved shear stress on each slip system. To consider the influence of these parameters on the accumulation of fatigue damage in materials from a statistical perspective, this paper proposes the following three sets of statistical parameters that account for mesoscale slip deformation and the resolved shear stress driving it (the first set contains 2 parameters, the second set contains 1 parameter, and the third set contains 2 parameters, for a total of 5 statistical parameters), to explore their evolution laws and apply them to the analysis of fatigue behavior and life prediction of polycrystalline pure copper.

1. The mean value and standard deviation of the sum of the absolute values of the resolved shear strains of all slip systems in each element within the RVE at the peak tensile points of the cycles. In Equation (13), *γ*^(*α*)^ represents the cumulated resolved shear strain for α-slip system.

(13) $$\Gamma =\sum_{\alpha =1}^{12}\left|\gamma^{\left(\alpha \right)}\right|,\;\gamma^{\left(\alpha \right)}=\int_{0}^{t}{\dot{\gamma }}^{\left(\alpha \right)}\mathrm{dt}\;\bar{\Gamma }=\sum_{k=1}^{n_{\mathrm{RVE}}}{\left(\Gamma \right)}_{k}p_{k}\;\;\hat{\Gamma }=\sqrt{\sum_{k=1}^{n_{\mathrm{RVE}}}{\left(\Gamma \right)}_{k}^{2}p_{k}-\bar{\Gamma }}\;$$

2. The standard deviation of the sum of the products of the resolved shear strains and resolved shear stresses of all slip systems in each element within the RVE at the peak tensile points of the cycles.

(14) $$\omega =\sum_{\alpha =1}^{12}\tau^{\left(\alpha \right)}\gamma^{\left(\alpha \right)}\;\bar{\omega }=\sum_{k=1}^{n_{\mathrm{RVE}}}{\left(\omega \right)}_{k}p_{k}\;\;\hat{\omega }=\sqrt{\sum_{k=1}^{n_{\mathrm{RVE}}}{\left(\omega \right)}_{k}^{2}p_{k}-\bar{\omega }}\;$$

3. The mean value and standard deviation of the product of the sum of the absolute values of the resolved shear stresses and the sum of the absolute values of the resolved shear strains of all slip systems in each element within the RVE at the peak tensile points of the cycles.

(15) $$\Xi =\left(\sum_{\alpha =1}^{12}\left|\gamma^{\left(\alpha \right)}\right|\right)\left(\sum_{\alpha =1}^{12}\left|\tau^{\left(\alpha \right)}\right|\right)\;\bar{\Xi }=\sum_{k=1}^{n_{\mathrm{RVE}}}{\left(\Xi \right)}_{k}p_{k}\;\hat{\Xi }=\sqrt{\sum_{k=1}^{n_{\mathrm{RVE}}}{\left(\Xi \right)}_{k}^{2}p_{k}-\bar{\Xi }}\;$$

In the above equation, *n_RVE_* is the total number of elements within the RVE; *γ*^(*α*)^ and *τ*^(*α*)^ are the shear strain and shear stress of the *α*-th slip system, respectively. The subscript *k* denotes the finite element index, *p_k_* = Δ*V_k_*/*V*, where Δ*V_k_* is the volume of the *k*-th element, and *V* is the total volume of the RVE. Therefore, *p_k_* is the relative volume fraction of the *k*-th element. Γ is the sum of shear strains of slip systems in the *k*-th element; $\bar{\Gamma }$ and $\hat{\Gamma }$ are the mean value and standard deviation of Γ statistics within the RVE, respectively; ω is the sum of the products of shear strain and shear stress of slip systems in the *k*-th element, and $\hat{\omega }$ is the standard deviation obtained from statistics of ω within the RVE; Ξ is the product of the sum of absolute shear strains and the sum of absolute shear stresses of slip systems in the *k*-th element, and $\bar{\Xi }$ and $\hat{\Xi }$ are the mean value and standard deviation of Ξ statistics within the RVE, respectively.

The selection of these three statistical parameters reflects the accumulation of microscopic damage and the resulting deformation inhomogeneity in polycrystalline metallic materials under fatigue loading, examined from three distinct perspectives: deformation geometry, energy dissipation, and stress–strain coupling. Specifically, the parameter Γ, based on the sum of the absolute values of accumulated shear strain across all slip systems, quantifies the degree of irreversible plastic deformation induced by dislocation motion and accumulation; high Γ values typically foreshadow the formation of persistent slip bands (PSBs) and surface extrusions/intrusions, which are precursors to micro-crack nucleation. The parameter ω quantifies plastic work through the sum of the products of resolved shear stress and resolved shear strain on each slip system, reflecting the driving effect of energy dissipation on the evolution of lattice defects such as vacancies and dislocation tangles; when the local energy dissipation density exceeds a critical threshold, damage accumulation is accelerated. Meanwhile, the parameter Ξ, derived from the product of the sums of absolute shear stress and shear strain, accounts for the synergistic effect between stress amplitude and deformation amplitude, effectively capturing the interaction between local stress concentration and macroscopic plastic deformation. Together, these three parameters construct a multidimensional damage characterization system spanning deformation magnitude, energy dissipation, and mechanical coupling, enabling a precise description of the gradual evolution and accumulation of microscopic damage in polycrystalline metallic materials during low-cycle fatigue from multiple perspectives.

In this paper, the above three parameters are taken as indicators to characterize the initiation of fatigue cracks, and three sets of FIPs are defined to investigate their evolution laws with the increase in cycle number. Assuming that material failure occurs when they reach a certain critical value, they can be expressed by the following formulas, respectively:

(16) $$\bar{\Gamma }={\bar{\Gamma }}_{\mathrm{fatigue}},\hat{\Gamma }={\hat{\Gamma }}_{\mathrm{fatigue}}$$

(17) $$\hat{\omega }={\hat{\omega }}_{\mathrm{fatigue}}$$

(18) $$\bar{\Xi }={\bar{\Xi }}_{\mathrm{fatigue}},\hat{\Xi }={\hat{\Xi }}_{\mathrm{fatigue}}$$

These three sets of indicator parameters can be used individually to determine the initiation of fatigue cracks and respectively provide predictions for the material’s fatigue life characteristic curves.

### 4.2. Statistical Characteristics of Crystal Slip-Based FIPs with Cyclic Variation

#### 4.2.1. Variations in Resolved Shear Strain and Resolved Shear Stress on the α-th Slip System (Taking the Peak Points of the 3rd and 498th Cycles at a Strain Amplitude of 0.006 as Examples)

**Figure 9:**
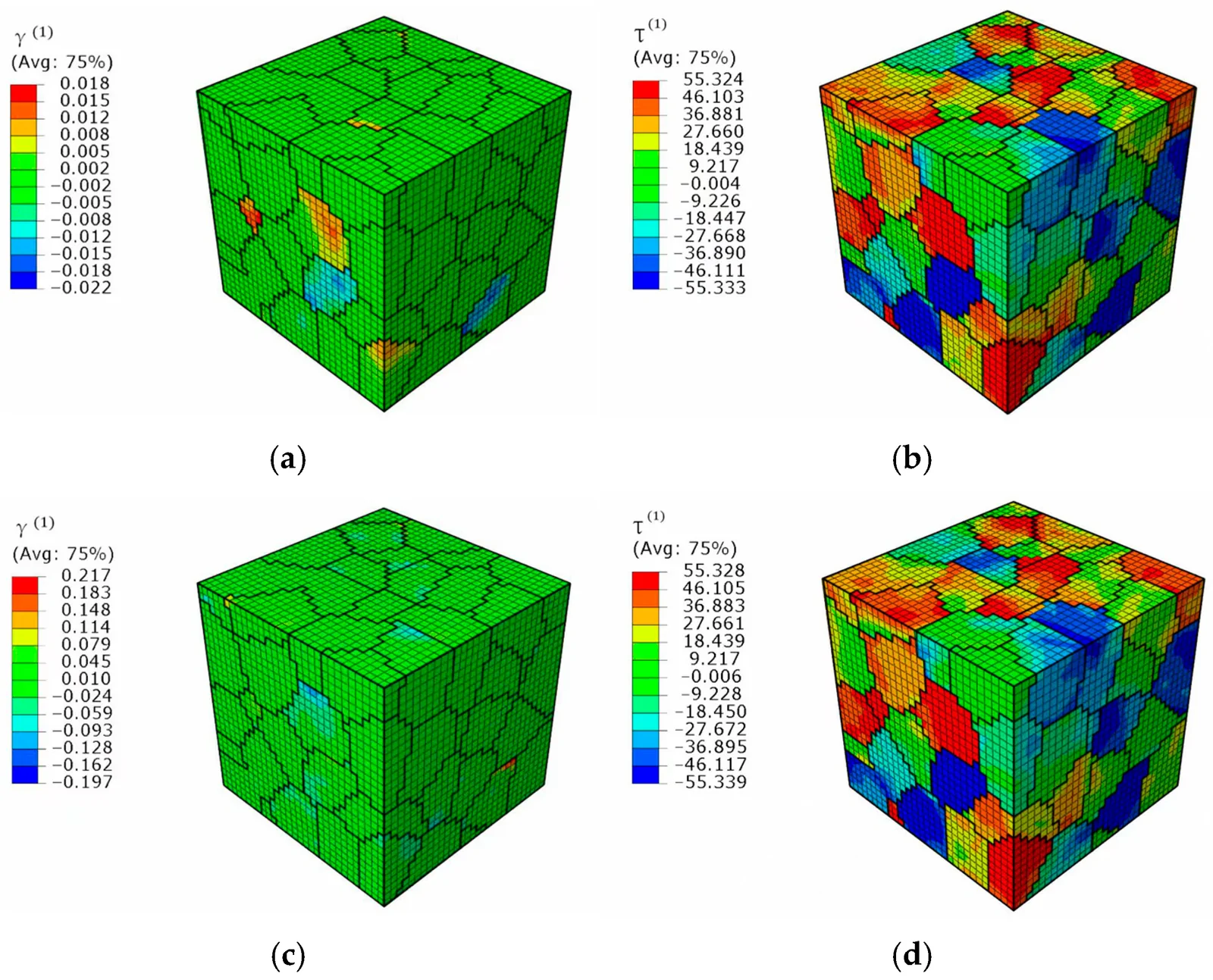
Contour plots of the resolved shear strain and resolved shear stress on the first slip system at the tensile peak points of the 3rd and 498th cycles under a strain amplitude of 0.006. (**a**) Contour plot of shear strain for the first slip system at the peak point of the 3rd cycle tensile loading; (**b**) Contour plot of shear stress on the first slip system at the peak point of the 3rd cycle tensile loading; (**c**) Contour plot of shear strain for the first slip system at the peak point of the 498th cycle tensile loading; (**d**) Contour plot of shear stress for the first slip system at the peak point of the 498th cycle tensile loading.

Due to the scatter in the mechanical behavior of materials at the mesoscale, a statistical description is needed to characterize the inhomogeneous mesoscopic deformation and its evolution with cycling, based on the RVE analysis. Taking the cyclic process at a strain amplitude of 0.006 as an example, as shown in Figure 9, the resolved shear strain difference in the first slip system (*α* = 1) at the 498th cycle (approaching 0.27 ${\bar{N}}_{f}$) increases significantly compared with that at the initial 3rd cycle, while the corresponding resolved shear stress distribution range shows no obvious change for the respective cycles. This is because, with the increase in cycle number, plastic deformation undergoes a strong heterogeneous evolution at the microstructural level, leading to a significant increase in local strain differences; whereas stress, as a response to strain, tends to saturate or change more slowly in the macroscopic amplitude under strain-controlled mode, and does not exhibit the severe local divergence as the local strain does. Therefore, slip deformation can be taken as the primary influential (or indicator) parameter for fatigue life prediction. Figure 9 shows the contour plots of the resolved shear strain and resolved shear stress of the first slip system, while the mesoscopic inhomogeneous deformation of the material is the result of the combined action of all slip systems. Therefore, the parameters for predicting fatigue life need to consider the resolved shear strains and resolved shear stresses of all slip systems.

#### 4.2.2. Evolution of FIPs with Cycle Number (Taking the Peak Points of the 3rd and 498th Cycles at a Strain Amplitude of 0.006 as Examples)

**Figure 10:**
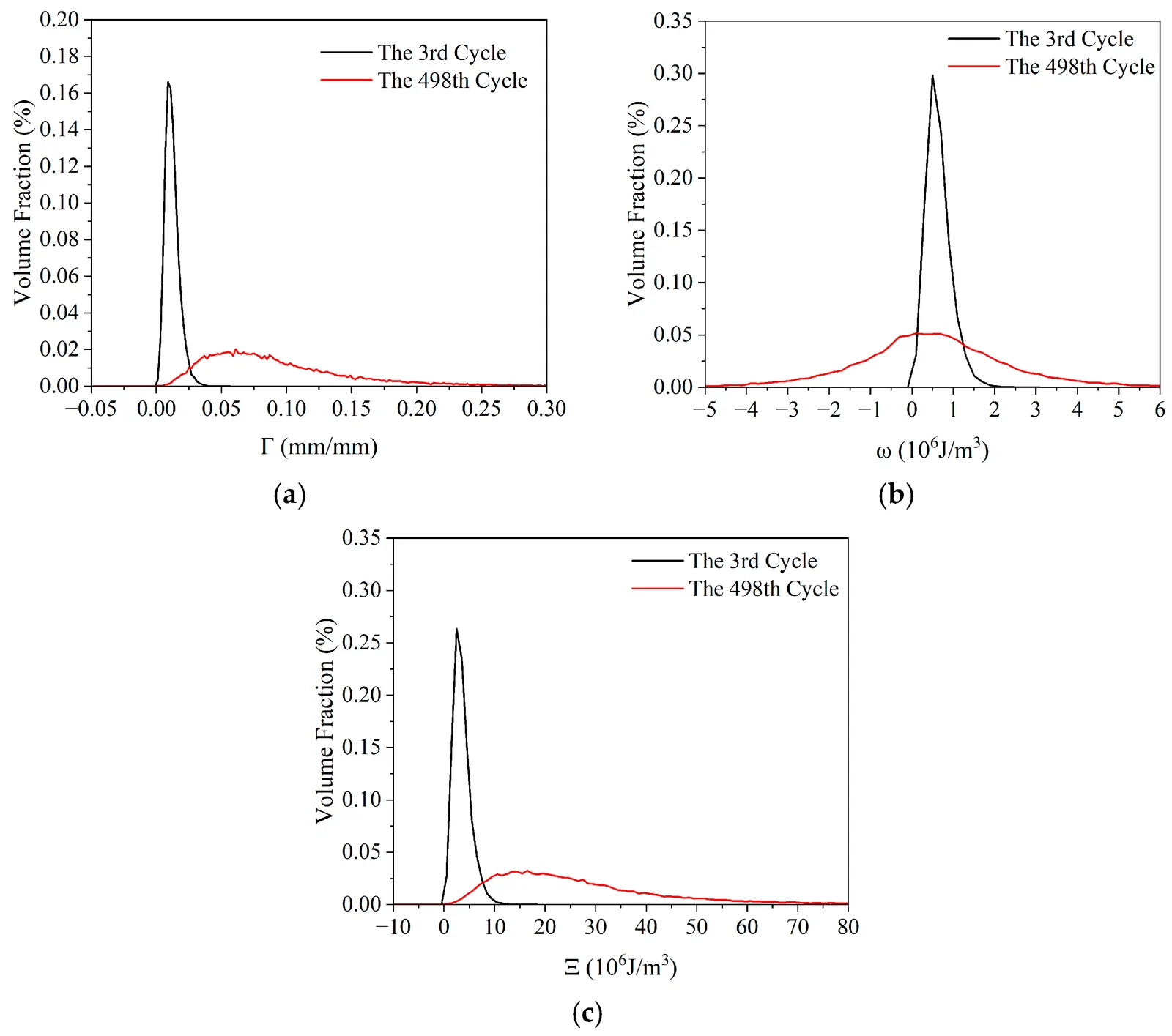
Distribution curves of Γ, ω and Ξ at the tensile peaks of the 3rd and 498th cycles when the strain amplitude is 0.006. (**a**) Distribution curves of Γ at the tensile peak of the 3rd and 498th cycles; (**b**) Distribution curves of ω at the tensile peak of the 3rd and 498th cycles; (**c**) Distribution curves of Ξ at the tensile peak of the 3rd and 498th cycles.

The statistical distribution curves of the indicator parameters Γ, ω, and Ξ defined in the previous text are plotted in Figure 10, which clearly shows that, compared with the 3rd cycle, the distribution range of the three sets of FIPs corresponding to the 498th cycle has increased by about 3 times, indicating that the statistical standard deviations of the three sets of statistical parameters have all increased. The centers of the distribution curves of Γ and Ξ significantly shift to the right after a certain number of cycles, indicating that the mean values of these two statistical parameters also increase; however, the increase in the mean value of ω is not obvious, implying that it is insensitive to the microstructural evolution of the material caused by fatigue, and therefore it is not used as a FIP.

#### 4.2.3. Evolution Laws of FIPs with Cycle Number

**Figure 11:**
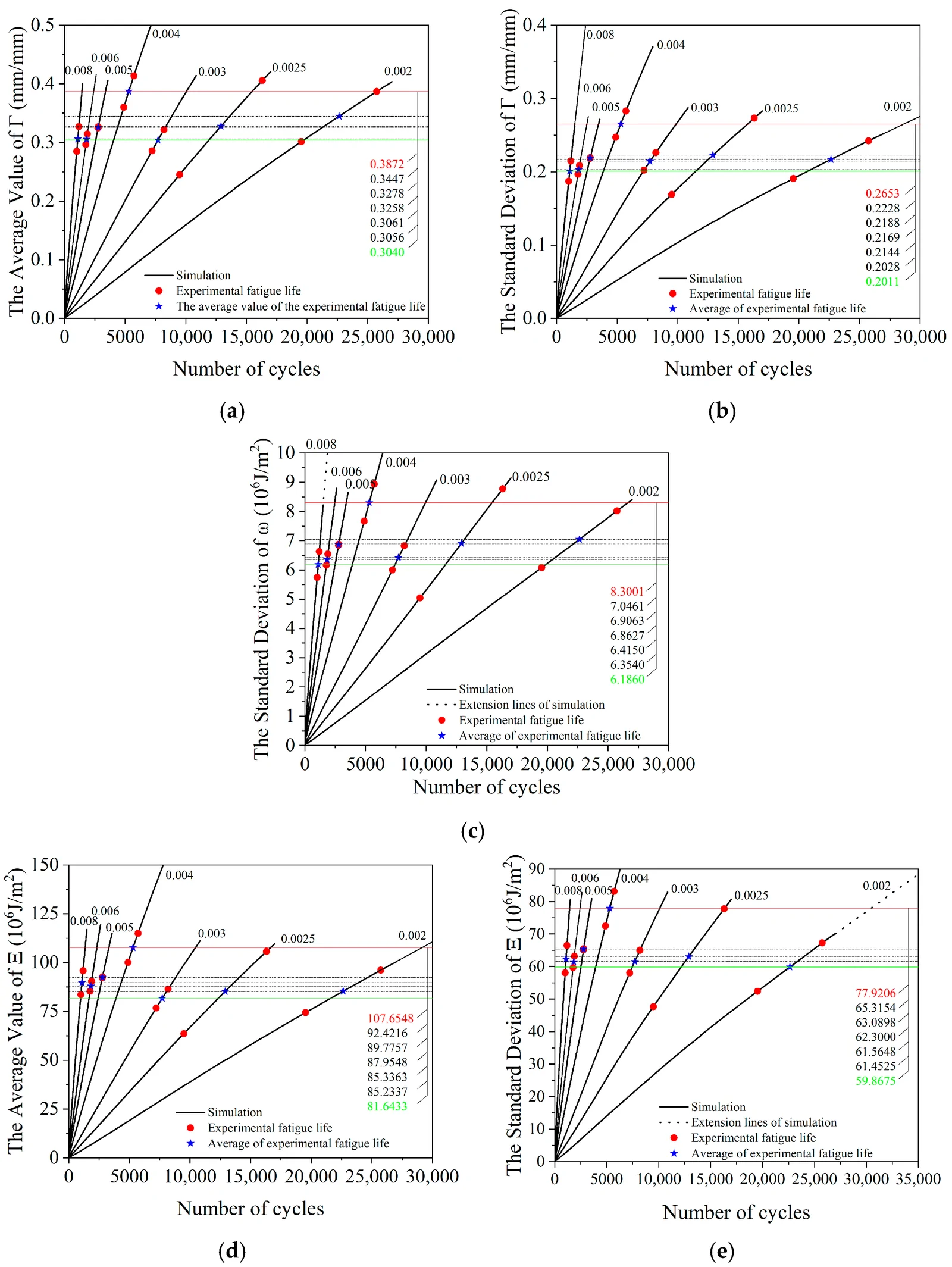
Evolution of heterogeneous slip deformation characterization parameters and critical values of FIPs for T2 pure copper under different strain amplitudes. (**a**) Evolution of $\bar{\Gamma }$ within RVE at tensile peak under different strain amplitudes with cycle number and critical value of ${\bar{\Gamma }}_{\mathrm{fatigue}}$; (**b**) Evolution of $\hat{\Gamma }$ within RVE at tensile peak under different strain amplitudes with cycle number and critical value of ${\hat{\Gamma }}_{\mathrm{fatigue}}$; (**c**) Evolution of $\hat{\omega }$ within RVE at tensile peak under different strain amplitudes with cycle number and critical value of ${\hat{\omega }}_{\mathrm{fatigue}}$; (**d**) Evolution of $\bar{\Xi }$ within RVE at tensile peak under different strain amplitudes with cycle number and critical value of ${\bar{\Xi }}_{\mathrm{fatigue}}$; (**e**) Evolution of $\hat{\Xi }$ within RVE at tensile peak under different strain amplitudes with cycle number and critical value of ${\hat{\Xi }}_{\mathrm{fatigue}}$. The red and green numbers indicate the maximum and minimum critical values of FIPs, respectively, while the black numbers represent the values in between.

By simulating the cyclic loading processes at strain amplitudes of 0.002, 0.0025, 0.003, 0.004, 0.005, 0.006, and 0.008, the variation curves of the mean value $\bar{\Gamma }$ and standard deviation $\hat{\Gamma }$ of the sum of absolute values of resolved shear strains of slip systems, the standard deviation $\hat{\omega }$ of the sum of products of resolved shear strains and resolved shear stresses, and the mean value $\bar{\Xi }$ and standard deviation $\hat{\Xi }$ of the product of the sum of absolute values of resolved shear strains and the sum of absolute values of resolved shear stresses at the cyclic peak points corresponding to each strain amplitude, with the cycle number *N*, can be obtained, as shown by the solid black lines in Figure 11. The curves in the figure show that under cyclic loading at different strain amplitudes, the above three sets of FIPs all increase with cycling, indicating that the slip deformation of the material becomes increasingly inhomogeneous. Moreover, after undergoing a certain number of cycles, all three sets of FIPs exhibit approximately linear growth. The calculations of $\bar{\Gamma }$ and $\hat{\Gamma }$, $\hat{\omega }$, $\bar{\Xi }$ and $\hat{\Xi }$ for the material are carried out until they exceed the larger of the two fatigue lives of pure copper obtained from experiments.

### 4.3. Fatigue Life Prediction Based on Inhomogeneous Deformation of Pure Copper

#### 4.3.1. Critical Values of Individual FIPs Under Different Strain Amplitudes

The experimental fatigue life data points of pure copper are plotted on the evolution curves in Figure 11, where the red solid circles represent individual measured fatigue lives, and the blue five-pointed stars represent the mean values of the measured fatigue lives. The y-axis coordinates of the measured average life points correspond to the critical values of the above-mentioned FIPs. Table 5 lists the critical values of $\bar{\Gamma }$ and $\hat{\Gamma }$, $\hat{\omega }$, $\bar{\Xi }$ and $\hat{\Xi }$ corresponding to the mean values of the measured fatigue lives of pure copper at different strain amplitudes. The slight scatter in the numerical values of fatigue indicator parameters is a normal phenomenon arising from experimental scatter. Each of the critical values in Table 5 can be used, together with the corresponding sub-figure curves in Figure 11, to determine the predicted fatigue life. The portions of $\hat{\omega }$ (at a strain amplitude of 0.008) and $\hat{\Xi }$ (at a strain amplitude of 0.002) that were not computed are extended with black dashed lines.

#### 4.3.2. Predicted Fatigue Life Obtained from Different Critical Values

**Figure 12:**
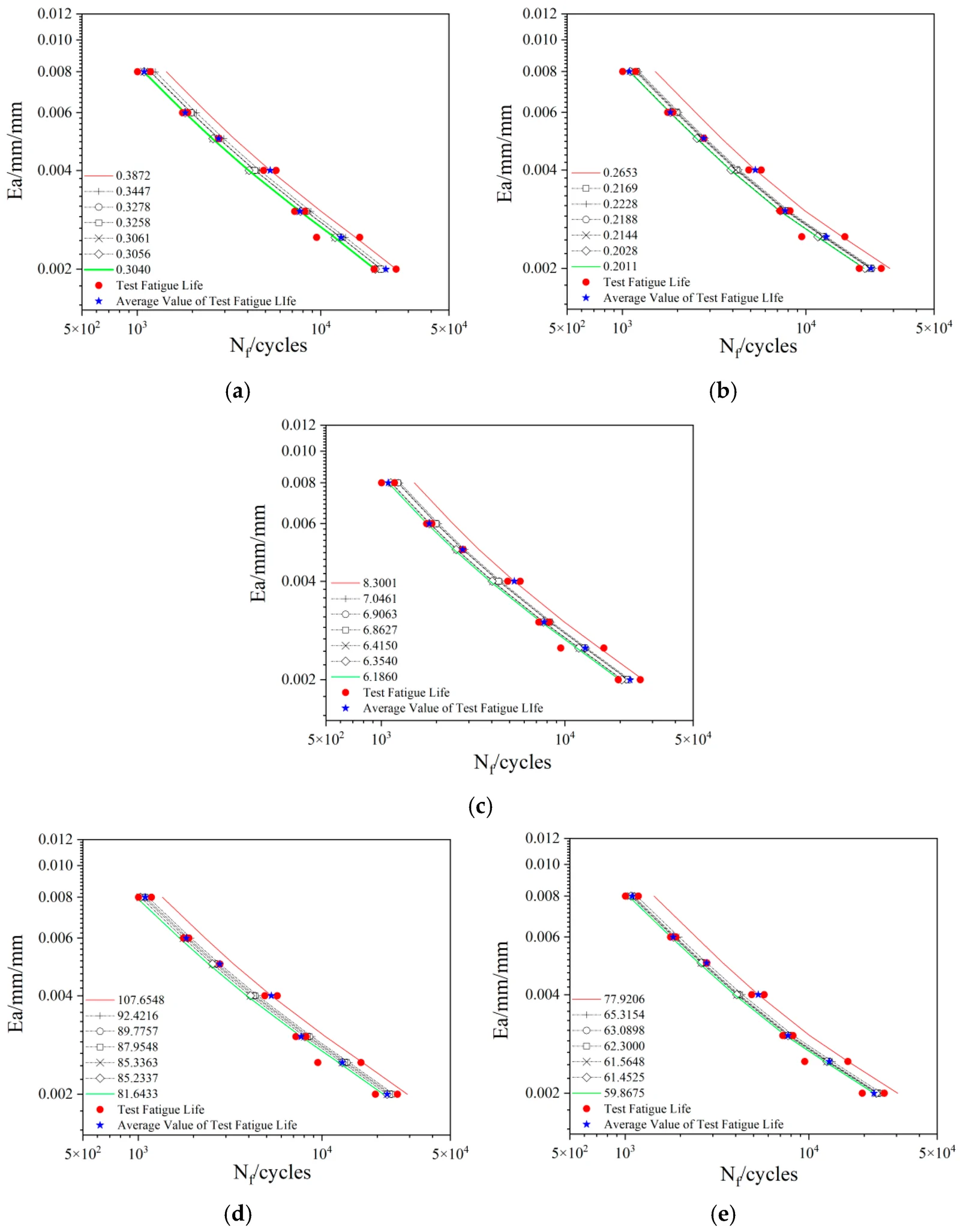
Family of predicted fatigue life curves for T2 pure copper obtained from the critical values of FIPs characterizing slip deformation inhomogeneity, and comparison with experimental life. (**a**) Family of predicted life curves obtained from the critical values of ${\bar{\Gamma }}_{\mathrm{fatigue}}$; (**b**) Family of predicted life curves obtained from the critical values of ${\hat{\Gamma }}_{\mathrm{fatigue}}$; (**c**) Family of predicted life curves obtained from the critical values of ${\hat{\omega }}_{\mathrm{fatigue}}$; (**d**) Family of predicted life curves obtained from the critical values of ${\bar{\Xi }}_{\mathrm{fatigue}}$; (**e**) Family of predicted life curves obtained from the critical values of ${\hat{\Xi }}_{\mathrm{fatigue}}$.

Taking ${\bar{\Gamma }}_{\mathrm{fatigue}}$ as an example to illustrate the life prediction process, each strain amplitude corresponds to a critical value of ${\bar{\Gamma }}_{\mathrm{fatigue}}$, with a total of 7 critical values. Each critical value can be used to predict the fatigue life at other amplitudes (see Table 6a), and each can also yield a corresponding critical fatigue prediction curve (see Figure 12a). Specifically, taking the critical value of ${\bar{\Gamma }}_{\mathrm{fatigue}}$ at a strain amplitude of 0.004, which is 0.3872, as an example, the predicted life in column 5 of Table 6a and the red life prediction curve in Figure 12a can be obtained. Since 0.3872 is the maximum value among the 7 critical values, the predicted life obtained from it is also the longest predicted life. Similarly, the predicted lives and life prediction curves corresponding to each critical value of the other FIPs $\hat{\Gamma }$, $\hat{\omega }$, $\bar{\Xi }$ and $\hat{\Xi }$ can be obtained (see Table 6 and Figure 12).

#### 4.3.3. Family of Predicted Fatigue Life Curves at Different Strain Amplitudes

Based on the critical values of the FIPs in Table 6, the family of predicted fatigue life curves under seven strain amplitudes can be obtained, as shown in Figure 12. In the figure, the red solid line and the green solid line represent the predicted fatigue life curves obtained from the maximum and minimum values of the FIPs, respectively, while the remaining black legends correspond to the prediction results obtained from the other critical values in Table 6. The predicted fatigue life curves agree well with the experimental fatigue life, which verifies the effectiveness of the low-cycle fatigue life prediction method proposed in this paper.

### 4.4. Error Evaluation of Predicted Fatigue Life for Pure Copper

**Figure 13:**
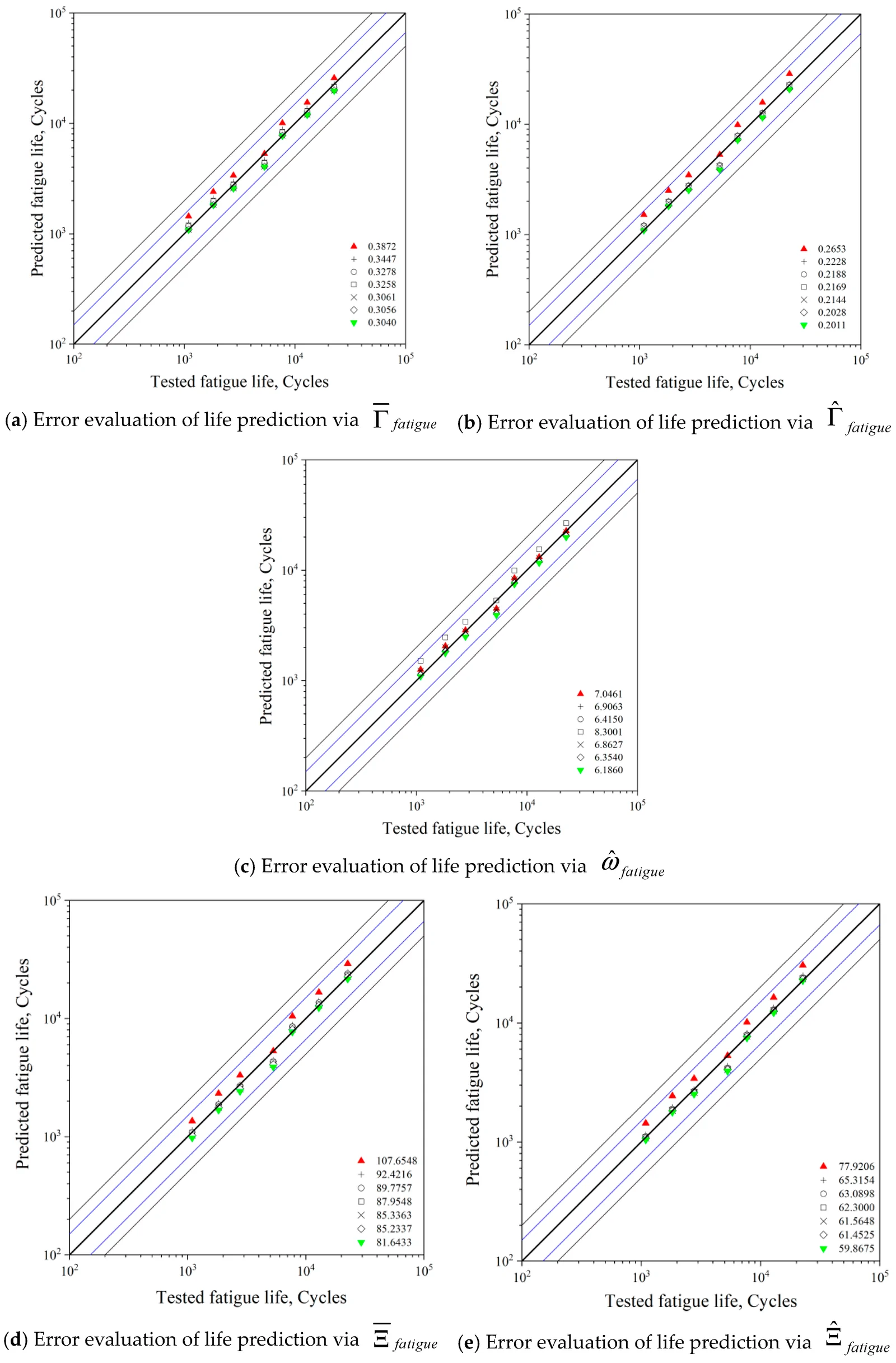
Error evaluation of predicted fatigue life versus measured life for T2 pure copper via FIPs characterizing slip deformation inhomogeneity.

To evaluate the rationality of the fatigue life prediction method described in this paper, the predicted life points are plotted within a 2-fold error band. As shown in Figure 13, the thick black solid line represents the ideal prediction, the region enclosed by the thin blue solid lines is the 1.5-fold error range, and the region enclosed by the thin black solid lines is the 2-fold error range. For the FIPs ${\bar{\Gamma }}_{\mathrm{fatigue}}$ and ${\hat{\Gamma }}_{\mathrm{fatigue}}$, ${\hat{\omega }}_{\mathrm{fatigue}}$, ${\bar{\Xi }}_{\mathrm{fatigue}}$ and ${\hat{\Xi }}_{\mathrm{fatigue}}$, regardless of whether the maximum or minimum value is taken, the resulting predicted life falls within the 1.5-fold error band. In contrast, the method proposed in this paper not only exhibits excellent prediction accuracy, with all predicted values falling within the ±1.5 scatter band and outperforming the 2-factor error band reported in the literature, but also possesses greater universality [40,41,42]. It enables life prediction across the full range of strain amplitudes by determining only a single critical FIP value, eliminating the need for complex regression fitting for each specific loading condition [60,61].

## 5. Conclusions

In this paper, a Voronoi RVE reflecting the heterogeneity of polycrystalline metallic materials and a crystal plasticity constitutive model considering anisotropic nonlinear kinematic hardening are adopted to numerically simulate the strain fatigue process of T2 pure copper. Based on the accurate reproduction of the macroscopic hysteresis behavior of pure copper, the methods for quantitatively characterizing the inhomogeneity of slip deformation under cyclic loading are deeply investigated, and new FIPs applicable to fatigue life prediction are proposed. The main conclusions are as follows:

(1) By combining the RVE with the crystal plasticity constitutive model, and based on the selection of reasonable parameters, the mechanical behavior of pure copper under symmetric strain cyclic loading is numerically simulated. The macroscopic hysteresis behavior of the material demonstrated by the simulation is consistent with the experimental observations.
(2) Three sets of FIPs based on slip system shear strain and stress statistics are proposed. These include the mean value $\bar{\Gamma }$ and standard deviation $\hat{\Gamma }$ of the sum of absolute values of slip system shear strains at the cyclic peak; the standard deviation $\hat{\omega }$ of the sum of products of slip system shear strain and shear stress; and the mean value $\bar{\Xi }$ and standard deviation $\hat{\Xi }$ of the product of the sum of absolute slip system shear strains and the sum of absolute shear stresses. These parameters can effectively characterize the inhomogeneity of internal slip deformation in the material. With the increase in cycles, the values of these parameters increase monotonically and exhibit a significant linear evolution relationship with the number of cycles in the middle and late stages of fatigue life.
(3) The critical values of the above three sets of FIPs at the time of fatigue failure can be utilized to predict the low-cycle fatigue life of pure copper. The predicted life curves obtained using different critical values show good agreement with the experimental data points, and all the predicted life results fall within a 1.5 times error band compared to the measured fatigue lives. Since the adopted constitutive model is based on the slip mechanisms of face-centered cubic (FCC) materials, it is capable of describing the mechanical behavior not only of pure copper but also of other FCC alloys. Consequently, the fatigue life prediction framework proposed in this study is applicable to other FCC materials.

## 6. Limitations and Future Perspectives

Although this study reveals the slip localization mechanism driven by intergranular interactions and the statistical laws of fatigue damage indicators through crystal plasticity finite element methods, certain limitations remain.

(1) To eliminate the interference of specific texture components and establish a baseline model that ignores preferred orientation, the Representative Volume Element (RVE) in this study adopted a random orientation assumption based on a uniform distribution of Euler angles, rather than directly incorporating experimentally measured EBSD initial crystallographic textures. For FCC metals like T2 copper, although random orientation models have been proven to reasonably describe macroscopic cyclic responses and the statistical trends of microscopic mechanical inhomogeneity, supplementing with real texture information could more accurately describe the evolution of fatigue parameters, thereby refining the framework of the life prediction method described in this paper.
(2) The validation of the current model is mainly limited to specific uniaxial loading conditions and does not yet cover complex stress states, which somewhat restricts the direct verification of the method’s universality. Meanwhile, this study did not investigate fatigue behavior under different strain ratios (R ≠ −1), nor did it include examinations of complex paths such as multiaxial or non-proportional loading. Future research will be dedicated to expanding the applicability of this method from the perspective of mechanical loading conditions.
(3) The validation of the simulated local deformation heterogeneity currently relies on the theoretical consistency of the crystal plasticity model, lacking direct experimental verification via techniques such as Digital Image Correlation (DIC), EBSD-based strain analysis, or slip-trace analysis. Although a comprehensive experimental comparison falls beyond the scope of this paper, future work will be dedicated to overcoming these technical hurdles to conduct in-situ observations, thereby providing robust experimental evidence to further validate the model’s predictions.

## Figures and Tables

**Table 1:** Chemical composition of industrial pure copper T2 (mass fraction) [45].

Chemical Composition/%											
Cu & Ag	P	Bi	Sb	As	Fe	Ni	Pb	Sn	Zn	Mn	Cd
99.935	0.0416	0.0036	0.001	0.0015	0.0032	0.0023	0.003	0.0019	0.0025	0.0034	0.001

**Table 2:** Basic mechanical properties of T2 pure copper.

E/MPa	*σ*_0.2_/MPa	*σ_b_*/MPa	*δ*/%	*ψ*/%	${\bar{\varepsilon }}_{f}$
110,800	30.1	199.6	57.5	71.1	1.24

**Table 3:** Uniaxial tension-compression cyclic fatigue life of T2 pure copper.

*E_a_*	*N_f_*	${\bar{N}}_{f}$	$E_{a}^{p}/\%$	${\bar{E}}_{a}^{p}/\%$
0.002	25,740/19,527	22,634	0.000978/0.000974	0.000976
0.0025	9493/16,309	12,901	0.00144/0.00142	0.00143
0.003	7215/8197	7706	0.00180/0.00179	0.001795
0.004	5716/4886	5301	0.00269/0.00272	0.002705
0.005	2770/2785	2778	0.00362/0.00356	0.00359
0.006	1767/1888	1828	0.00445/0.00450	0.004475
0.008	1004/1180	1092	0.00623/0.00629	0.00626

**Table 4:** Crystal plastic model parameters for T2 copper.

*C*_11_	*C*_12_	*C*_44_	*τ*_0_	*τ_s_*	*h*_0_	*a*	*c*	*p*	*e*_1_	*e*_2_	${\dot{\gamma }}_{0}$	*q*	*k*
GPa	GPa	GPa	MPa	MPa	MPa	GPa	GPa	s^−1^			s^−1^		
124.6	98.3	61.1	29.8	30	66.4	30	1.18	0	0	0	0.001	1	200

**Table 5:** Critical value of FIPs $\bar{\Gamma }$ and $\hat{\Gamma }$, $\hat{\omega }$, $\bar{\Xi }$ and $\hat{\Xi }$ corresponding to the mean experimental fatigue life under different strain amplitudes for T2 copper.

*E_a_*	Average Test Life	${\bar{\Gamma }}_{\mathrm{fatigue}}$	${\hat{\Gamma }}_{\mathrm{fatigue}}$	${\hat{\omega }}_{\mathrm{fatigue}}$	${\bar{\Xi }}_{\mathrm{fatigue}}$	${\hat{\Xi }}_{\mathrm{fatigue}}$
0.002	22,634	0.3447	0.2169	7.0461	85.3363	59.8675
0.0025	12,901	0.3278	0.2228	6.9063	85.23373	63.0898
0.003	7706	0.3040	0.2144	6.4150	81.6433	61.5648
0.004	5301	0.3872	0.2653	8.3001	107.6548	77.9206
0.005	2778	0.3258	0.2188	6.8627	92.4216	65.3154
0.006	1828	0.3056	0.2028	6.3540	87.9548	61.4525
0.008	1092	0.3061	0.2011	6.1860	89.7757	62.3000

**Table 6:** (a) Predicted fatigue life obtained from the critical value of ${\bar{\Gamma }}_{\mathrm{fatigue}}$ within the RVE. (b) Predicted fatigue life obtained from the critical value of ${\hat{\Gamma }}_{\mathrm{fatigue}}$ within the RVE. (c) Predicted fatigue life obtained from the critical value of ${\hat{\omega }}_{\mathrm{fatigue}}$ within the RVE. (d) Predicted fatigue life obtained from the critical value of ${\bar{\Xi }}_{\mathrm{fatigue}}$ within the RVE. (e) Predicted fatigue life obtained from the critical value of ${\hat{\Xi }}_{\mathrm{fatigue}}$ within the RVE.

(a)							
$\bar{\Gamma }$	**Ea = 0.002** **(0.3447)**	**Ea = 0.0025 (0.3278)**	**Ea = 0.003** **(0.3040)**	**Ea = 0.004** **(0.3872)**	**Ea = 0.005** **(0.3258)**	**Ea = 0.006** **(0.3056)**	**Ea = 0.008** **(0.3061)**
*E_a_*	*N_f_*						
0.002	22,634	21,410	19,698	25,758	21,266	19,817	19,851
0.0025	13,620	12,901	11,902	15,473	12,817	11,971	11,991
0.003	8836	8363	7706	10,055	8307	7751	7764
0.004	4653	4399	4046	5301	4369	4070	4077
0.005	2959	2797	2570	3378	2778	2586	2590
0.006	2099	1980	1816	2403	1966	1828	1831
0.008	1255	1183	1083	1439	1175	1090	1092
(b)							
$\hat{\Gamma }$	**Ea = 0.002** **(0.2169)**	**Ea = 0.0025 (0.2228)**	**Ea = 0.003** **(0.2144)**	**Ea = 0.004** **(0.2653)**	**Ea = 0.005** **(0.2188)**	**Ea = 0.006** **(0.2028)**	**Ea = 0.008** **(0.2011)**
*E_a_*	*N_f_*						
0.002	22,634	23,345	22,329	28,655	22,859	20,948	20,743
0.0025	12,515	12,901	12,352	15,765	12,637	11,611	11,500
0.003	7810	8053	7706	9869	7887	7239	7169
0.004	4209	4339	4154	53,014	4251	3903	3866
0.005	2751	2835	2715	3464	2778	2552	2527
0.006	1975	2038	1948	2505	1995	1828	1810
0.008	1193	1231	1177	1512	1205	1103	1092
(c)							
$\hat{\omega }$	**Ea = 0.002** **(7.0461)**	**Ea = 0.0025 (6.9063)**	**Ea = 0.003** **(6.414979)**	**Ea = 0.004** **(8.3001)**	**Ea = 0.005** **(6.8627)**	**Ea = 0.006** **(6.3540)**	**Ea = 0.008** **(6.1860)**
*E_a_*	*N_f_*						
0.002	22,634	22,188	20,609	26,645	22,049	20,412	19,867
0.0025	13,153	12,901	12,013	15,434	12,822	11,902	11,598
0.003	8457	8290	7706	9947	8238	7634	7434
0.004	4475	4383	4058	5301	4354	4018	3907
0.005	2856	2796	2586	3406	27,787	2559	2487
0.006	2051	2006	1847	2457	1992	1828	1774
0.008	1263	1235	1138	15,131	1226	1126	1092
(d)							
$\bar{\Xi }$	**Ea = 0.002** **(85.3363)**	**Ea = 0.0025 (85.2337)**	**Ea = 0.003** **(81.6433)**	**Ea = 0.004** **(107.6548)**	**Ea = 0.005** **(92.4216)**	**Ea = 0.006** **(87.9548)**	**Ea = 0.008** **(89.7757)**
*E_a_*	*N_f_*						
0.002	22,634	22,605	21,582	29,126	24,664	23,383	23,904
0.0025	12,918	12,901	12,325	16,639	14,070	13,341	13,637
0.003	8082	8072	7706	10,453	8817	8352	8541
0.004	4087	4082	3892	5301	4466	4227	4324
0.005	2539	2535	2416	33,056	2778	2627	2688
0.006	1765	1763	1678	2316	1935	1828	1871
0.008	1029	1027	976	1355	1130	1066	1092
(e)							
$\hat{\Xi }$	**Ea = 0.002** **(59.8675)**	**Ea = 0.0025 (63.0898)**	**Ea = 0.003** **(61.5648)**	**Ea = 0.004** **(77.9206)**	**Ea = 0.005** **(65.3154)**	**Ea = 0.006** **(61.4525)**	**Ea = 0.008** **(62.3000)**
*E_a_*	N_f_						
0.002	22,634	23,985	23,347	30,423	24,913	23,300	23,656
0.0025	12,177	12,902	12,556	16,339	13,410	12,531	12,722
0.003	7469	7922	7706	10,105	8240	7690	7810
0.004	3935	4174	4061	5301	4340	4052	4115
0.005	2517	2672	2599	3405	2779	2593	2634
0.006	1776	1886	1834	2433	1965	1830	1859
0.008	1043	1109	1078	1436	1156	1075	1093

## Data Availability

The original contributions presented in this study are included in the article. Further inquiries can be directed to the corresponding authors.
